# Exercise or lie down? The impact of fitness app use on users' wellbeing

**DOI:** 10.3389/fpubh.2023.1281323

**Published:** 2024-01-10

**Authors:** Jie Cai, Gang Li

**Affiliations:** School of Sports, Shandong University of Finance and Economics, Jinan, China

**Keywords:** fitness app, exercise, lie down, upward social comparison, self-control

## Abstract

**Introduction:**

The use of fitness apps is becoming more and more widespread, and its impact on people's well-being has received more and more attention.

**Methods:**

The relationship between fitness app use and users' well-being and the influence mechanism was explored using structural equation modeling with upward social comparison as the mediating variable and self-control as the moderating variable.

**Results:**

The questionnaire survey of 1,452 fitness app users over 18 years old shows that: (1) fitness app use is associated with users' well-being; (2) upward social comparison plays a mediating role in the relationship between fitness app use and users' well-being; (3) self-control has a moderating effect on the relationship between fitness app use and users' well-being.

**Discussion:**

Self-control plays a significant moderating role between social comparison and well-being, upward social comparison can improve the well-being of high self-control users but reduce the well-being of low self-control users.

## 1 Introduction

Fitness apps offer fitness training classes, store users' exercise and fitness data, and provide recommendations for healthy lifestyles (1). Fitness apps can improve the regularity of users' physical activities, the frequency of fitness exercises and walking behavior (2). Long-term use of fitness app will improve people' s physical, emotional, social, and cognitive status and promote their wellbeing (3). The different social impacts of fitness apps appeal to different types of users. Although fitness apps have brought many benefits in promoting health and have been widely recognized, the negative effects of fitness apps have received little attention (4).

In fitness marketing, social comparison, especially upward social comparison, is often used as a stimulus and marketers promote the application by showing the perfect body image in fitness apps. Marketers believe that by seeing near-perfect body images in advertisements, consumers will feel dissatisfied with their bodies and will be motivated to improve their body image by using fitness products. The influence of upward social comparison of body images on fitness app user behavior has been extensively studied. It has been found that upward social comparison may discourages users from exercise training, lead to body anxiety (5) and lower self-esteem (6). In contrast, some studies pointed out that upward social comparison could promotes users to strengthen self-discipline and makes them feel happy (7). So it is unclear whether upward social comparison motivate users to actively participate in exercise, making them happy or make them reluctant to engage in fitness training, choose to lie down, feel inferior or depressed.

It is biased to analyze the impact of upward social comparison on user behavior from a single negative or positive perspective. Self-control can improve people's happiness levels, and people who are good at self-control are more likely to experience happiness and be more satisfied with their lives, self-control has emphatically corresponded with happiness (8). Self-control and social comparison have an interaction in predicting older person's wellbeing (9). But whether the wellbeing of fitness app users with different self-control capacities varies in the upward social comparison process is still worthy of being explored. In the present study, based on the social comparison theory and self-control theory, we discussed the impact of fitness app use on users' wellbeing in the Chinese context and the influence mechanism. This paper enriches the research on fitness apps. It is of reference significance to improve users' wellbeing and promote the long-term development of fitness apps.

## 2 Theoretical background and research hypothesis

### 2.1 The influence of fitness app use on users' wellbeing

The new media represented by the Internet and mobile phones have become an important factor affecting the daily lifestyle of the public. As a kind of sports media, fitness apps can promote users' exercise and fitness behaviors based on big data technology by motion trajectory tracking, goal setting, feedback, reward, social connection and remote guidance (10). Fitness apps have grown exponentially during COVID-19 lockdowns (11). Currently, the vast majority of sports services offer their users fitness apps to facilitate aspects such as communication and information (12). Studies are increasing as well as a specifical interest in fitness apps (13). Fitness apps can provide users with feedback information on diet, exercise, and body condition, improving physical fitness levels, which can significantly affect wellbeing (14). Compared with traditional gyms, fitness apps cost less. Long-term use will improve people's physical, emotional, social, and cognitive status and promote their wellbeing (3). Mobile health (mHealth) app positively influences users' perceived wellbeing, users rate higher levels of wellbeing regarding the mHealth app before use than after use (15). Using fitness apps allow users to engage in constant self-tracking and are described as a way to practice body awareness, it was found that fitness app use and wellbeing has causal relationship (1). Fitness apps can improve the regularity of users' physical activities, the frequency of fitness exercises and walking behavior, and enhance their wellbeing (2). Fitness app use, body awareness, and wellbeing have a close relationship through the MARS (Motivation-Ability-Role-perceptions-Situational Factors) model (1). Based on the above findings, hypothesis 1 is established as follows.

H1: Fitness app use is associated with users' wellbeing.

### 2.2 The mediating role of upward social comparison

People are surrounded by a large amount of information every day in the big data era. People are more inclined to know themselves by comparing themselves with those perceived to be better in their circles. This process is called upward social comparison (16). People often make upward social comparisons consciously or subconsciously with being overexposed to social media information (17). Individuals compare others' ability, achievements, wealth and appearance with themselves by browsing online information in network socializing, resulting in upward social comparison activity (18). Social comparison help humans navigate social groups by providing them with information about their own relative standing (19). Although upward social comparison has some benefits, always comparing yourself to someone who is doing better than you is thought to cause some potential problems (20). Users show positive self-bias online than in real life, so it is thought that negative social comparisons are more likely to occur on social media (21). They may particularly elicit envy, “the pain caused by the good fortune of others,” which can impair wellbeing (22). Studies have shown that lower social status/rank is associated with more psychological and behavioral problems. Upward social comparison not only generates negative emotional perception of one's own social status, but also stimulates subsequent behavioral problems (23).

At present, fitness apps have richer content and more diverse forms, and the impact of upward social comparison on the relationship between app use and wellbeing should be investigated. Many scholars have pointed out that fitness apps may promote upward social comparison among users. For example, it has been found that the display of body images and self-discipline behaviors in fitness apps encourages users to actively exercise and control calorie intake, which causes upward social comparison in terms of living habits and fitness behaviors (7). Fitness app users who engaged in upward fitness comparison had elevated motivation and participation in PA, especially it makes them feel happy (24). Some researchers found that self-presentation in fitness apps stimulates users' interest in sports and motivates them to show their exercise and fitness achievements to compete with others, improve users' wellbeing (25). However, some scholars have pointed out its negative effects. Exposure to images of idealized bodies in the media has been shown to increase body dissatisfaction among women. One of the mechanisms through which exposure influences body dissatisfaction is appearance-based comparison with the people in the images (26). The photos women see on social networking sites such as Instagram and Facebook may also create and reinforce their unrealistic ideals (27, 28). App usage increases engagement in social comparison which influence users' happiness, social comparison fully mediates the effect of app-use on happiness (29). Based on the above findings, hypothesis 2 is established as follows.

H2: Upward social comparison mediates the relation between fitness app use and wellbeing.

### 2.3 The moderating role of self-control

Self-control (SC) is the process or behavior by which an individual is able to resist temptation or place more importance on (simultaneous or long-term) goal achievement (30). How does self-control affect an individual's sense of wellbeing? Related research has not provided a definite answer to this question yet. One view is that self-control may require frequent suppression of unrealistic emotions, thoughts, and behaviors, leading to a life dominated by stereotypes and banality, which reduces wellbeing (31). Another view is that self-control could improve individual wellbeing (32). Studies have found that people with strong self-control experience closer relationships (33). People with high self-control report higher levels of wellbeing (34), including better psychological adjustment, higher levels of wellbeing (35) and higher life satisfaction (34), are more likely to achieve wellbeing (36). A self-control meta-analysis confirmed that people with high trait self-control showed good adaptability in work, school, interpersonal relationships and health (37). Users with lower self-control have lower wellbeing when using SNS, whereas users with high self-control have higher wellbeing (38). Self-control can play a moderating role, and people with high self-control generally have better health and higher life satisfaction (39). Individuals' self-control would improve subjective wellbeing, and self-control mediate the relation between social support and subjective wellbeing (40). Individuals' self-control and social comparison have an interaction in predicting wellbeing: individuals with high self-control have a stronger positive prediction of wellbeing by social comparison (9). It can be concluded that there is a close relationship between good self-control and individual wellbeing (32). Based on the above analysis, hypothesis 3 is established.

H3: Self-control moderates the relationship between upward social comparison and users' wellbeing.

**Figure 1:**
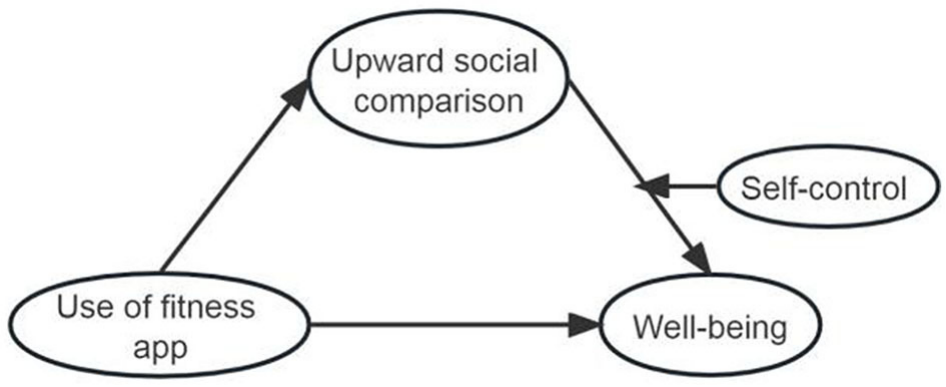
Structural model.

Based on the above hypotheses, the structural model is shown in Figure 1.

## 3 Materials and methods

### 3.1 Sampling and participants

The questionnaire survey method was used to collect data. In accordance with the principle of convenience and effectiveness, we adopted the method of intentional sampling to recruit respondents from several QQ groups and WeChat groups of fitness enthusiasts. In order to ensure the validity of the questionnaire survey, 117 users were selected for a pre-test before the formal distribution of questionnaires, and all of the questionnaires were retrieved, 108 of which were valid. Due to the impact of COVID-19, the questionnaires were distributed online. The study was approved by the Ethics Committee of Sports in SDUFE (2022/003, January 17, 2022) and was conducted following the Declaration of Helsinki established by the World Medical Association. The informed consent of the participants was obtained at the beginning of the survey. During the formal survey, in order to improve the enthusiasm of the participants and the quality of their answers, they were told in advance that they would get 5 RMB as a reward if they answered the questions in the questionnaire earnestly. A total of 1,500 questionnaires were distributed through an online Chinese survey platform (https://www.wjx.cn), a lie detector question is set in the questionnaire, that is, “I have never used fitness app.” If the respondent choose Yes, it means that the respondent did not answer the questionnaire carefully, and the answer is invalid. The electronic questionnaire can show the respondent's answer time, if the answer time is <1 min, the answer is invalid. Finally, 1,452 valid questionnaires were collected.

Through the analysis of valid samples, it was found that 815 were women (69.7%) and 637 were men (30.3%); 24.3% were aged 18–25, 44.2% were aged 26–35, 20.4% were aged 36–45, and 11.1% were aged 46 or older. Among them, 55.8% went to college, 17.7% had a master's or higher degree, and 26.5% only had a high school diploma or a lower educational level. In addition, 673 (38.3%) had high self-control, and 779 people (61.7%) had low self-control. Young women with higher education levels were the main users of fitness apps.

### 3.2 Research tool

All the variables except self-control were evaluated using the Likert5-level scale, with 1–5 points indicating “strongly disagree” to “strongly agree.” The measurement items of all variables were determined based on previous research results and were adjusted according to the fitness app.

The questionnaire consists of variable measurement questions and demographic questions. The variable measurement items were designed according to the existing mature scale, mainly including fitness app use, upward social comparison, wellbeing and self-control.

#### 3.2.1 Fitness app use

The fitness app use scale developed by Kagkini (41) was adopted. Five items were selected and slightly modified: (1) I use fitness app to track activity; (2) I set personalized exercise goals with fitness app; (3) I ensure meeting exercise goals by using fitness app; (4) I participate or compete with others with the app; (5) I strive to earn digital rewards.

#### 3.2.2 Upward social comparison

The upward social comparison scale compiled by Lee (42) was used, and four items were determined: (1) I often think that others have a happier life, when I see their when I read their exercise news feeds or see photos or videos of them exercising; (2) I often think that others have a better life when I read their exercise news feeds or see photos or videos of them exercising; (3) I often think that others have a healthier life when I read their exercise news feeds; (4) I often think that others feel better than me when I read their exercise news feeds or see photos or videos of them exercising.

#### 3.2.3 Users' wellbeing

The wellbeing scale developed by Diener et al. (43) was adopted, and five items were determined: (1) in most ways my life is close to my ideal; (2) the conditions of my life are excellent; (3) I am satisfied with my life; (4) so far I have gotten the important things I want in life; (5) if I could live my life over, I would change almost nothing.

#### 3.2.4 Self-control

The evaluation of self-control was performed mainly based on the brief self-control scale (44), and the item was slightly modified: If the respondent is offered a 30 RMB coupon for any purchase over 50 RMB, they can choose between food apps and fitness apps. Choosing fitness apps means high self-control, and choosing food apps means low self-control. The choice tendency of the respondents was measured (1 = fitness app, 0 = food app).

## 4 Results

Structural equation modeling (SEM) was use to assess the structural model, SEM can model the proposed relationship between several predictors of unobjectable potential variables and standard variables with flexibility (45). Relate latent variables to observable variables through measurement models. In addition, SEM has no measurement error in analyzing the relationship between mental structures, and can also analyze the dependence between latent variables (46). The Bootstrap method was used to test the mediating mechanism of upward social comparison and the moderating effect of self-control.

### 4.1 Reliability and validity analysis

Table 1 summarizes the evaluation results of the structural model. The reliability and the convergent validity and discriminative validity of all constructs passed the test. Cronbach's alpha (0.809–0.870) indicates that the internal consistency of construction items is good. The composite reliability of constructs (0.886–0.916) indicates high validity. Average variance extracted (AVE; 0.637–0.687) demonstrating that convergent validity was satisfactory. Squared multiple correlations (AVE; 0.415–0.824) >0.36 indicated the reliability of the items was good. Factor loading ranged from 0.630 to 0.908 and was significant at 0.001. The discriminant validity test results for latent variable are shown in Table 2. The variables have high discriminant validity because the square root of AVE value of the latent variable was greater than the absolute value of its correlation coefficient with other latent variables.

**Table 1:** Variable factor load/reliability and validity indexes.

**Latent variable**	**Items**	**Estimate**	**SMC**	**CR**	**AVE**	**Cronbah's α**
Fitness app use (FI)	Track activity	0.893	0.797	0.896	0.637	0.856
	Set personalized exercise goals	0.837	0.701			
	Ensue meeting exercise goals	0.908	0.824			
	Participate or compete with others with the app	0.644	0.415			
	Strive to earn digital rewards	0.668	0.446			
Upward social comparison (UP)	I often think that others have a happier life	0.807	0.651	0.886	0.665	0.809
	I often think that others have a better life	0.897	0.805			
	I often think that others have a healthier life	0.899	0.808			
	I often think that others feel better than me	0.630	0.397			
Wellbeing (WE)	In most ways my life is close to my ideal	0.898	0.806	0.916	0.687	0.870
	The conditions of my life are excellent	0.872	0.760			
	I am satisfied with my life	0.895	0.801			
	So far i have gotten the important things i want in life	0.752	0.566			
	If i could live my life over, i would change almost nothing.	0.707	0.500			

**Table 2:** Test table for the discriminant validity of variables.

	**AVE**	**FI**	**UP**	**WE**
FI	0.637	**0.798**		
UP	0.665	0.528	**0.815**	
WE	0.687	0.456	0.507	**0.829**

Values in bold represent the square root of AVE (average variance extracted) values.

### 4.2 Direct effect test of hypothesis

The results of the predictive accuracy assessment of the model was shown in Table 3. According to the fitting index of SEM, the 90% confidence interval for the RMSEA (0.053; 0.067) is acceptable as values under 0.08 are deemed acceptable. Both CFI and TLI are >0.9, χ^2^2/DF is <3, both of which met the test criteria of fitting indicators, and had appropriate predictive ability for each endogenous variable, indicating that the model met the fitting requirements. Table 3 also shows the results of the measurement model assessment. Testing the direct effects after excluding non-significant paths indicated that fitness app use was related to users' wellbeing providing support for H1, respectively. This shows that the use of fitness app is associated with the user's wellbeing, and the user's wellbeing will be improved with the use of fitness app.

**Table 3:** Parameter estimation and hypothesis testing of analytical models.

**Path**	**Estimated value**	***P*-value**
Wellbeing ← Fitness app use	0.261	^***^
Upward social comparison ← Fitness app use	0.528	^***^
Wellbeing ← Upward social comparison	0.369	^***^
Fit index: χ^2^/df = 2.442; CFI = 0.976; TLI = 0.970		

*** *P* < 0.001.

### 4.3 Mediating effect test

According to Table 3, fitness app use can positively predict upward social comparison and wellbeing, respectively. Therefore, mplus7.4 software was used to test the mediating effect of the structural model. After adding the mediating variable of upward social comparison into the prediction model of fitness app use on wellbeing, fitness app still positively predicts wellbeing, and upward social comparison positively predicts wellbeing. The test results of the mediating effect of upward social comparison are shown in Table 4. The mediating effect was significant within a 95% confidence interval after 1,000 Bootstraps. Generally speaking, when the bias-corrected confidence interval and percentile confidence interval do not contain 0, the mediating effect exists. Through the test of the mediating effect of upward social comparison, H2 is empirically supported, the mediating effect was 0.194. This suggests that fitness app use can be associated with users' wellbeing through upward social comparison.

**Table 4:** Test results of mediating effect.

**Mediating route**	**Point estimate**	**Product of co-efficients**			**Bootstrap 1,000 times 95% CI**			
					**Bias-corrected**		**Percentile**	
		**S.E**.	**Est./S.E**	***P*** **-value**	**Lower 2.5%**	**Upper 2.5%**	**Lower 2.5%**	**Upper 2.5%**
Wellbeing ← Upward social comparison ← Fitness app-use	0.195	0.031	6.382	^***^	0.107	0.247	0.113	0.257

*** *P* < 0.001.

### 4.4 Moderating effect test

The results of the moderating effect of high/low self-control are shown in Table 5. The results showed that the moderating effect was significant within the 95% confidence interval after 1,000 Bootstraps.

**Table 5:** Test results of moderating effect for high/low self-control.

**New/additional parameters**	**Bootstrap 1,000 times 95% CI**	
	**Lower 2.5%**	**Upper 2.5%**
FIWE1	0.181	0.731
FIWE2	0.155	0.394
FIWE1-FIWE2	0.020	0.710

To clarify the moderating effects of self-control on the relationship between upward social comparison and wellbeing, Table 6 lists the non-standardized path coefficients of the group model. In the two models, the path coefficient of the high self-control group was 0.431 (*P* < 0.001), and the path coefficient of the low self-control group was 0.180 (*P* < 0.05). This indicates that high self-control users have a stronger feeling of wellbeing in the process of upward social comparison than that low self-control users. H3 is empirically supported.

**Table 6:** Path coefficient of grouping model.

**Path co-efficient**	**High self-control (*N* = 673)**	**Low self-control (*N* = 779)**
Wellbeing ← Upward social comparison	0.431	0.180

So far, the three hypotheses proposed in this study have been empirically tested. The effect of fitness app use on wellbeing is partly mediated by upward social comparison, with self-control tendency moderating the latter half of this mediating effect.

## 5 Discussion

### 5.1 Relationship between fitness-app use and users' wellbeing

First of all, this study fills the gap in the theoretical basis of users' wellbeing in the field of fitness app research. Fitness app use was related to users' wellbeing, and users can improve their wellbeing by using fitness apps. This verifies the view that physical activity can improve people's wellbeing (47) and that, as a kind of social media, fitness apps can promote people's wellbeing (48). Fitness app users will feel happy during physical activities (2); users who exercise for a longer time and more frequently will have fewer negative emotions; users who insist on exercise will have better physical and mental health conditions and experience more wellbeing flow (1). When users are completely immersed in exercise and fitness training, their psychological pressure and anxiety from work, life or family can be reduced, and their wellbeing can be improved (49). The present study showed that fitness apps users who enjoy exercising or engaging in fitness training and exchanging exercise information would get more wellbeing (13). The research results confirm that fitness apps provide users with a platform to share exercise experiences and show fitness achievements, which promotes social communication (3); in this way, the users can temporarily take their minds off their troubles and relieve themselves from anxiety and depression (11). It can be concluded that using fitness apps can improve users' wellbeing. In the marketing of fitness apps, attention should be paid to stimulating users' cognition that “I can do what others can do.” For example, as the most famous fitness APP in China, KEEP has set up a section called “My Story,” which encourages users to record the changes in their bodies during the use of the app. Their stories can motivate others to think that through self-control, they can also build a strong body, have an excellent body image, and gain a sense of freedom and wellbeing.

### 5.2 The mediating role of upward social comparison

Upward social comparison plays a mediating role between fitness app use and wellbeing. Previous studies have shown that upward social comparison plays a mediating role in the relationship between social app use and users' satisfaction (50), this study further confirms the results of this study. Users compare others' ability, achievements, wealth and appearance with themselves in network socializing, resulting in upward comparison activity (18). However, the influence mechanism of fitness app use on users' wellbeing has not been revealed, and how upward social comparison plays a mediating role remains to be investigated. The present study takes upward social comparison as the mediating variable and empirically verifies the mediating effect of upward social comparison on fitness app use and users' wellbeing. The findings indicate that fitness app directly affects users' wellbeing (51); it also indirectly affects wellbeing through upward social comparison. In addition, most previous studies have focused on the negative consequences of upward social comparison. People's dissatisfaction with their body image is a manifestation of their self-control failure in the process of upward social comparison (26). By demonstrating the relationship between upward social comparison and users' wellbeing, this study proves the research hypotheses and defines scenarios in which upward social comparison has positive or negative effects on fitness app users. Fitness apps should take social responsibility. During daily marketing activities such as advertising, the impact of upward social comparison on users' physical and mental health should be considered. Extreme social comparisons should be avoided to prevent self-abasement. Fitness apps should focus on helping users maintain healthy physical and mental states and improve their wellbeing.

### 5.3 The moderating role of self-control

This study also verifies the hypothesis that self-control is a moderating variable of the relationship between upward social comparison and wellbeing. It can be seen that self-control is a complex psychological concept. When individuals are in the upward social comparison environment, users with different self-control abilities will have completely different psychological states. After the upward social comparison, high self-control users had a higher sense of wellbeing than lower self-control users (9). The possible reasons may be as follows. High self-control users will regard the gap between them and those that they believe are better as an opportunity for self-improvement and a direction for their efforts (35). Therefore, they will be more willing to take positive actions and exercise harder in upward social comparison, and they will gain wellbeing in the process of continuous self-improvement. In the upward social comparison process, users with high self-control would generate a belief of “I can also reach this state through hard exercise,” which will constantly motivate them to achieve the goal, enhance their confidence and anti-frustration ability, and improve their sense of self-efficacy. In this process, they can improve their self-body satisfaction and will feel happy (32). In contrast, low self-control users will experience negative emotions like self-doubt and fall into body anxiety (38). They are more likely to feel frustrated about the huge gap between their own body image and the perfect body image, which strengthens the threat effect generated by upward social comparison (5). The uncontrollability generated in this process will cause body anxiety (50). It reduces the sense of self-efficacy and causes low self-control users to choose to “lie down” to avoid this threat source, which reduces their wellbeing. Afterward, they may succumb to short-term indulgence to avoid fitness training and even give up using fitness apps. It can be summarized that compared with low self-control users, high self-control users have a higher level of wellbeing when involved in the same upward social comparison.

## 6 Implications and limitations

The current results contribute to the literature on fitness app in several ways. Firstly, these findings have enriched and improved the theoretical system of fitness app use. Most previous studies only focused on the wellbeing (2) and body anxiety (26) brought about by the use of fitness apps, whereas ignoring the mediating role of upward social comparison. Secondly, our study reveals that self-control plays a significant moderating role between social comparison and wellbeing. Previous studies on fitness app have focused mostly on positive results brought by apps (3, 14). Our study found that people with different self-control have different results when using apps. Finally, this study provides theoretical and empirical basis for the development of fitness apps, which is conducive to in-depth understanding of the connotation of upward social comparison and self-control, and helps individuals and platforms to actively and effectively deal with psychological adaptation problems caused by upward social comparison.

Further, the findings from this study have implications for the fitness app platform, this study further distinguishes situations in which fitness app use predicting users' wellbeing as mediated by upward social comparison with different self-control capacities. Irrational behaviors such as giving up fitness can help people regain self-worth in a short time, but in the long term, users will feel guilty or regretful and will become unhappy. Fitness apps can use big data technology to collect fitness data to create user portraits and accordingly provide personalized marketing information for users with different levels of self-control. For high self-control users, fitness apps should use upward social comparison as a marketing stimulus to motivate them to exercise and improve their body image and wellbeing. For users with low self-control, fitness apps should use conservative and prudent marketing methods to keep them away from self-threat or anxiety.

However, several limitations of this study should be noted. This study has the following limitations. Firstly, only online questionnaires were sent out, this may result in the majority of the interviewees being young people. Secondly, factors such as age and gender may influence the results of the study, and these factors should be included in the model in future studies. Despite its shortcomings, this study deepens the understanding of fitness app and provides user experience insights for app designers and marketers. Based on this, future studies can further explore the universality of questionnaires by enriching sample groups, continue to explore other paths and conditions that fitness app use is associated with users' wellbeing, and reveal a more comprehensive and detailed mechanism. Finally, future studies can also focus on the process and mechanism of downward social comparison on users' wellbeing, so as to more comprehensively understand the influencing mechanism of fitness app users' wellbeing.

## 7 Conclusion

This study discusses the influence of fitness app use on users' wellbeing. The results show that fitness app use is associated with users' wellbeing, with upward social comparison playing a mediating role and self-control playing a moderating role. Future researchers and practitioners can expand the sample size. Longitudinal studies can be conducted on other psychological impacts of fitness app use.

## Data availability statement

The original contributions presented in the study are included in the article/Supplementary material, further inquiries can be directed to the corresponding author.

## Author contributions

JC: Writing – original draft, Data curation, Investigation. GL: Conceptualization, Formal analysis, Writing – review & editing.
