# Supplementary material for: Exercise or lie down? The impact of fitness app use on users' wellbeing
Source: Front Public Health. 2024 Jan 10;11:1281323. doi: 10.3389/fpubh.2023.1281323 (PMC10806118; doi:10.3389/fpubh.2023.1281323)
Supplement: Supplementary file 1 [file Data_Sheet_1.docx]

Appendix. Survey questions and response options

**Measure of fitness app use**

We would like to know some things about your fitness app use. Please indicate how much you agree or disagree with each of the following statements. [For each statement, respondents rate their subjective agreement using a conventional five-point Likert Scale: 1 “Strongly disagree’’; 2 “Disagree”; 3 “Neither disagree nor agree”; 4 “Agree”; or 5 “Strongly Agree”. ]

1. I use fitness app to track activity.
2. I set personalized exercise goals with fitness app.

(3) I ensure meeting exercise goals by using fitness app.

(4) I participate or compete with others with the app.

(5) I have never used fitness APP.(R)

(6) I strive to earn digital rewards.

**Measure of upward social comparison**

We would like to know how you feel about others on fitness app. Please indicate how much you agree or disagree with each of the following statements. [For each statement, respondents rate their subjective agreement using a conventional five-point Likert Scale: 1 “Strongly disagree’’; 2 “Disagree”; 3 “Neither disagree nor agree”; 4 “Agree”; or 5 “Strongly Agree”. ]

1. I often think that others have a happier life, when I see their when I read their exercise news feeds or see photos or videos of them exercising.
2. I often think that others have a better life when I read their exercise news feeds or see photos or videos of them exercising.
3. I often think that others have a healthier life when I read their exercise news feeds. (4) I often think that others feel better than me when I read their exercise news feeds or see photos or videos of them exercising.

**Measure of well-being**

We would like you to evaluate your well-being with your own life. Please indicate how much you agree or disagree with each of the following statements. [For each statement, respondents rate their subjective agreement using a conventional five-point Likert Scale: 1 “Strongly disagree’’; 2 “Disagree”; 3 “Neither disagree nor agree”; 4 “Agree”; or 5 “Strongly Agree”. ]

1. In most ways my life is close to my ideal.
2. The conditions of my life are excellent.
3. I am satisfied with my life.
4. So far I have gotten the important things I want in life.

(5) If I could live my life over, I would change almost nothing.

**Measure of self-control**

If there was a 30 RMB coupon for any purchase over 50 RMB, one for food apps and one for fitness apps. Which one would you like to choose? (Single Choice)

A.Food app

B.Fitness app

**Measures for socio-demographics**

[For gender] What is your gender?

- Female

- Male

[For age] What is your age?

1. 18 to 25

2. 26 to 35

3. 36 to 45

4. 46 or older.

[For education level] What is the highest degree or level of education that you have completed?

1. Less than middle school

2. High school graduate

3. College or Bachelor's degree

4. Master degree

6. Doctor degree
